# Built Environment: Does Poor Housing Raise Diabetes Risk?

**DOI:** 10.1289/ehp.115-a534

**Published:** 2007-11

**Authors:** Adrian Burton

Poor housing may increase the risk of developing diabetes mellitus among middle-aged black Americans, suggests research published in the 15 August 2007 edition of the *American Journal of Epidemiology*. What exactly causes this link, however, remains to be explained.

“Many factors linked with the development of diabetes, such as obesity or the use of alcohol or smoking, are commonly present in the lives of people living in poorer housing,” explains first author Mario Schootman, chief of the Division of Health Behavior Research at Washington University in St. Louis, Missouri. But when his team adjusted for these factors, living in a poorly maintained house remained a significant risk for diabetes in its own right. In contrast, the quality of the neighborhood overall was not associated with increased risk.

According to the American Diabetes Association, diabetes already affects some 7% of the U.S. population and 13.3% of non-Hispanic blacks, with 90% of these cases comprising type 2 diabetes. The prevalence of diabetes could double by 2050—even triple among blacks, says Schootman. To better understand why this is so, the environmental context in which individuals live, work, and play needs to be taken into account, but until now no work has focused on the effect of neighborhood and housing conditions, factors that have been associated with other health problems including depression.

The researchers interviewed 644 middle-aged subjects enrolled in the African American Health Study who lived in either a poor inner-city area of St. Louis or a less impoverished suburb of the city. At the time of initial interview, no subject declared having been diagnosed with diabetes (the interviewers did not ask specifically about type 1, type 2, or other types of diabetes), although 10.3% went on to develop some form of the problem within three years.

During the first interviews the researchers took note of the respondents’ neighborhood and personal housing conditions. Neighborhoods were rated on a four-point scale ranging from excellent to poor depending on the general condition of the houses, the amount of ambient noise, general air quality, the state of repair of the streets, and other factors. Individual respondents’ housing conditions were similarly rated, on the basis of the physical condition of the interior, cleanliness, and the quality of the furnishings.

“We then looked for an association between these conditions and the development of diabetes among the study subjects,” explains Schootman, “and found every housing condition rated fair to poor, as well as the overall housing rating, to be associated with around a doubled risk of developing [diabetes].”

When the researchers used regression analysis to identify factors that might mediate this association—including household income, level of education, marital status, social support, access to medical care, health behaviors, body mass index, hypertension, number of medications used, and many other possibilities—none was found to be responsible.

Hilary Thomson, a research scientist at the Social and Public Health Sciences Unit of the U.K. Medical Research Council, remarks, “The authors have provided a thorough analysis and critique of the data, and it is difficult to think of additional important factors that might be responsible for this association, although it would be interesting to know of any interactions between the pathways. It would also be interesting to know what sort of interventions could be recommended.”

“Better housing variable scores, which are associated with a reduced odds ratio for developing diabetes, may identify resilient families who not only work to maintain their homes, but have good diets and exercise regularly,” suggests Philippa Howden-Chapman, program director of the Housing and Health Research Programme at the University of Otago in Wellington, New Zealand. “Also, housing attributes may be important, whether multilevel inner-city apartments or stand-alone suburban houses, in influencing availability of fresh food and places to exercise.”

Schootman agrees that the situation is complex. Whether other ethnic groups living in poor housing conditions face the same problem remains to be seen.

## Figures and Tables

**Figure:**
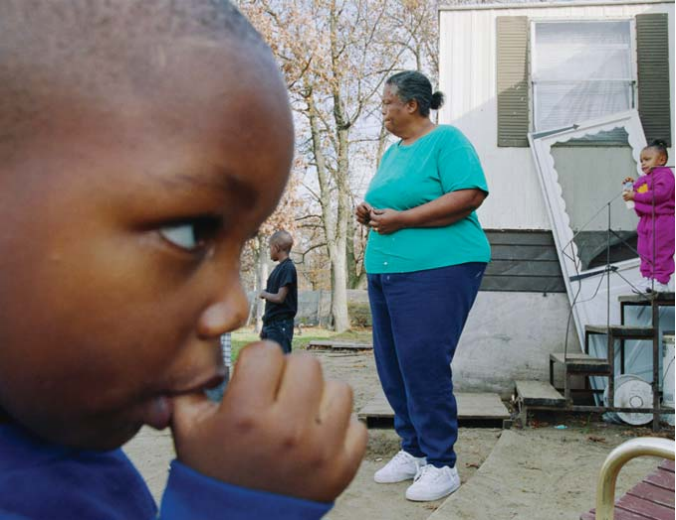
Scope of influence With diabetes possibly tripling among black Americans in the next two decades, it is essential to understand the full range of risk factors, which a new study suggests may include housing.

